# Typhoid Fever; Its Unusual Prevalence in the City of Chicago, Etc.

**Published:** 1882-02

**Authors:** N. S. Davis

**Affiliations:** Professor of Principles and Practice of Medicine and of Clinical Medicine in Chicago Medical College; Chicago


CHICAGO MEDICAL

JOURNAL & EXAMINER.

VOL. XLIV.—FEBRUARY, 1882.—NO. 2.

### Clinical Lectures.

### ARTICLE I.

TYPHOID FEVER: ITS UNUSUAL PREVALENCE IN THE CITY OF CHICAGO DURING THE SUMMER AND AUTUMN OF 1881; ITS CAUSES, CHARACTERISTICS AND TREATMENT. The substance of a Clinic in the Medical Wards of the Mercy Hospital, Jan. 20, 1882. By N. S. DAVIS, M.D., Professor of Principles and Practice of Medicine and of Clinical Medicine in Chicago Medical College.

GENTLEMEN: At the opening of the present college term the last week in September, you found an unusual number of cases of severe typhoid fever in the medical wards of this hospital, and your attention was occupied during almost every medical clinic-day in a bed-side study of cases of that important variety of fever for five or six weeks. I then had occasion to remark to you that the disease had been much more prevalent in this city during the summer and early autumn than the average of a term of years, and that the progress of individual cases was characterized by unusual severity. During the last month (December)

you have seen the number of cases steadily diminish in our wards until there are but few left, while their places have come to be occupied by a larger proportion of rheumatic, catarrhal, and pulmonary affections. Among the number left, is one, in the progress of which there are some points of interest to which I wish to direct your attention at the present time. Before I do so, however, I wish to present some facts that may explain, in part at least, the unusual prevalence of typhoid fever the past season. To assure myself of the correctness of my impression as to the extraordinary prevalence of the disease, I took the time to compare the official reports of mortality by the Health Department of the city for the nine months commencing April 1 and ending December 31, of 1880, with the same months of 1881. That you may appreciate the comparison better I will place it in tabular form before you on the blackboard, as follows:

| Number of Deaths from Typhoid Fever in— | 1880. | 1881. |
|---|---|---|
| April | 11 | 10 |
| May | 8 | 29 |
| June | 9 | 24 |
| July | 11 | 23 |
| August | 21 | 98 |
| September | 25 | 94 |
| October | 31 | 123 |
| November | 14 | 73 |
| December | 13 | 53 |
| Total | 143 | 527 |

You see by the figures that the total number of deaths from typhoid fever for the nine months of 1880 was 143, and the total number for the same months of 1881 was 527, being an increase of nearly four-fold. If we might suppose the mortality from this disease in 1880 was a little below the ordinary average, there would still remain sufficient increase to justify the appellation of epidemic for the disease in 1881. One of the most interesting and important questions suggested by the unusual prevalence of typhoid fever during the past season relates to the cause or causes that may have produced it. I presume no one will claim that there has been any fresh importation of fever germs or poison from abroad; neither can it be truthfully claimed that the sanitary condition of the city, so far as the accumulations of dirt and filth in the streets and alleys, and the conditions of sewers, etc., are concerned, was in any material degree worse in 1881 than in the preceding year. If this is conceded, then we must examine the meteorological characteristics of the two seasons to see if they afford any reasonable explanation.

In comparing the mean temperature of the nine months, I find that of 1880 to be 55.4° F., and that of 1881 to be 58.3° F. The mean for the months of April, May and June, 1880, was much higher than for the same months in 1881. The means for July and August were very nearly the same in both years, while the mean for each of the remaining four months was much higher in 1881 than in 1880. You see, therefore, that the higher aggregate mean temperature for the nine months of 1881 is owing entirely to the higher temperature of the last four months. But the figures in the table show that the unusual prevalence of fever commenced in May, when the mean temperature of that month was nearly 4° F. below that of the corresponding month of the preceding year; consequently we get no explanation from this comparison of mean temperatures. A comparison of the mean humidity and average rain-fall affords no more satisfactory results. And yet a more detailed study of the meteorological conditions accompanying the two years develops differences of a striking and important character. For instance, in the months of February and March of 1880, there was no unusual fall of either snow or rain, and April commenced with about the usual temperature and soil moisture, and throughout that and the succeeding month there were frequent rains, making the aggregate of fresh fallen water a full average for both months. The same was true in regard to the temperature and rain-fall for the next five months, from the first of June to the end of October. There was no protracted season of dry weather until November and December, when the temperature had become low. On the other hand, during the months of February and March, 1881, there was an extraordinary fall of snow, more especially during the last half of March. The snow fell in this region several feet deep. Its melting caused the month of April to be characterized by extraordinary floods over nearly the whole Northwest, and the soil was saturated to an unusual depth. Yet directly following the overwhelming saturation from the melting snows of March there were sixty-seven days during which there was hardly enough rain-fall to wet the dust in the streets. The first rain-fall of importance after the beginning of April was on the 6th, 7th and 8th of June. Then followed forty-two days more, until the 21st of July, without rain sufficient to penetrate the soil an inch at any one time. A liberal rain fell on the 21st of July, but no more of importance until the 15th of September, a period of fifty-four days. We thus find five and a half months of dry weather, broken by only two copious rains, and these separated by an interval of forty-two days, following a period of most complete saturation, and the last half of the time accompanied by a temperature favorable for both decomposition and evaporation of the morbid products. Another coincident item of much importance was the greater *stillness* of the atmosphere during all the months under consideration in 1881, compared with those of 1880. The aggregate number of miles travelled by the winds or atmospheric currents, at the government signal service station in this city, for the nine months of 1881 was 50,422; and for the same months of 1880 it was 56,779.

Not only was the aggregate distance traveled by the atmospheric currents much less in 1881, but the maximum velocity attained by the winds was from four to fifteen miles per hour less for each month, except September, which was four miles per hour more than in the corresponding month of 1880. By this study of the meteorological records, I have evolved three factors, or special characteristics, of much importance in their relations to the prevalence of typhoid fever. They are, first, the extreme saturation of the soil from melting snow during the first half of April; second, the long periods of dryness during the succeeding five months favoring the constant evaporation of the products of decomposition from the surface and sub-soil into the atmosphere; and third, the constant stillness of the atmosphere, which greatly favored the retention of the evaporated miasms in the lower strata, where they would be most readily inhaled by the community. That these coincident conditions were the chief agencies in causing the unusual prevalence and severity of typhoid fever during the past season, I have no doubt. This conclusion is further supported by the fact that there was, during the same time, a greater development of that form of malaria which produces periodical fevers, as shown by the marked increase in the mixed cases, called typho-malarial. Thus we have for nine months 65 deaths reported as from typho-malarial fever in 1881, and only 17 for the same period in 1880. Then a careful noting of the several data will show that the conditions which I have pointed out as efficiently favoring the prevalence of the fever, existed in their greatest intensity from the 21st of July to the middle of September, and if you remember that most of the deaths resulting from the typhoid form of fever occur during the fourth and fifth weeks after their commencement, you will see why the highest mortality was reached in October, and why the more copious rains, and greater prevalence of north and northeast winds in October, so rapidly diminished the number of attacks as to cause a great decline in the mortality for the following month of November. During the earlier part of the present college term, while calling your attention to the symptoms and treatment of several cases of typhoid fever in these wards, I stated that fever when used as the name of an acute general disease was something more than mere increased heat; that the latter was, indeed, only one of a number of important symptoms, resulting from a disturbance of the elementary properties of the tissues and of the molecular movements generally; and, consequently, that the prevailing antipyretic doctrine and practice were founded upon a mere symptom common to many diseases, instead of resting upon the actual pathological processes which constitute a distinct variety of fever.

As proof of the correctness of this view, I directed your attention to the fact, fully demonstrated by clinical experience, that the heat returned just as often as the influence of the antipyretic was withheld, until the disease had run its natural course. The correctness of these views has received striking confirmation by the facts embraced in a "Clinical Lecture on the Antipyretic Treatment of Typhoid Fever by Baths, Sponging the Body, and the Wet Sheet," in the Bellevue Hospital, by Austin Flint, M.D., etc., and published in a recent number of the *Philadelphia Medical News.* In this lecture Dr. Flint gives an interesting analysis of the treatment of seventeen cases in the wards of the hospital under his own direction during the present season, detailed records of which were kept by his assistants. The treatment consisted mainly in the employment of antipyretics, including the cold bath, wet sheet with sprinkling, and the sponge bath ; and in a less degree, sulphate of quinia and alcoholic liquids. Among the conclusions arrived at by the lecturer are, first, that by the employment of the external application of cold water in typhoid fever the temperature of the body can be reduced to 102° F. or lower. Second, that after a variable period of time the temperature, "as a rule, again rises as high as, or higher than, before the reduction." Third, that the cases analyzed "do not afford proof either that the fatality of typhoid fever or its duration is diminished" by the antipyretic measures employed. Of the seventeen cases mentioned as treated by the measures alluded to, four died.

While appreciating fully the value of frequent sponging of the cutaneous surface with water, whenever it is hot and dry, as a palliative in typhoid fever, I have found it to exert but a feeble influence over the progress of those general deteriorative molecular changes throughout both the organized structures and the organic constituents of the blood, which constitute the essential pathology of this variety of fever. To more effectually counteract these morbid changes we need some remedy capable of exerting a general alterant and antiseptic influence, and maintaining it for a considerable time without depressing the strength, or creating local complications. The last time I took you to the bedside of typhoid fever patients I called your attention to the effects of iodine, which I had then commenced giving, with the hope that it might be found capable of exerting more nearly the actual alterant and antiseptic influence needed, than any of the remedies hitherto used in such cases. Since then I have continued to use the remedy in all the well marked cases of typhoid coming under my supervision, both in the hospital and in private practice. Without counting the case before us to-day, which is yet under treatment, the whole number of well marked cases in which the iodine was given as the leading remedy, is fourteen. Seven of these cases occurred in private practice, and the other seven were treated in these wards. Of the seven cases treated outside of the hospital, five came under my care during the first three days after the patients took to their beds ; the other two not until the first half of the second week. Of those treated in the hospital, two were admitted on the third day of the fever, two on the fifth and sixth days, and the remaining three between the seventh and tenth after the commencement of the disease. You will note that nine of the fourteen cases were brought under treatment during the first week after the onset of the disease, and the other five not until the first half of the second week. The treatment in all these cases consisted in the administration of from 12 to 15 minims of the following solution of iodine :

℞ Iodinii .........0.5 grams ...................grs. viij
Potassii iodidi ......... 2.0 grams........ " xxx
Aquæ distillatæ ..........45.0 cc..........℥jss
M.

These doses were generally diluted with 30 cc. or two tablespoonfuls of sweetened water, and repeated every four hours for the first three or four days, and then every six hours until indications of convalescence appeared. Whenever the intestinal evacuations become too frequent and thin, a teaspoonful of the ordinary turpentine and laudanum emulsion was given between the doses of iodine. When the temperature rose to 40° C. (104° F.), and the skin dry, the patients were frequently sponged with cold water. Two of the seven treated in private practice took two grains of sulphate of quinia three times a day during the last week of their progress. Nearly all of the seven treated in the hospital wards took small quantities of the mineral acids largely diluted with water during the earlier part of their treatment, and small doses of quinine three or four times in the twenty-four hours during the latter part. All the fourteen were carefully nourished by the faithful giving of milk, wheat-flour and milk-gruel, and beef-tea, at regular intervals.

No alcoholic liquids, either fermented or distilled, were given to any of these patients during any part of their treatment. Of the nine cases in which the treatment was commenced during the first week after the patients took to their beds, four convalesced between the twelfth and fourteenth days; three between the fourteenth and seventeenth, and two between the seventeenth and nineteenth. Of the five cases in which the treatment was not commenced until the first half of the second week of their progress, three convalesced between the eighteenth and twenty-first days, and the other two between the twenty-first and the twenty-fifth. No one of the fourteen suffered a relapse, and no case terminated fatally. I now call your attention to the fifteenth case, in room 11, which was admitted into the hospital on the 3d of December, presenting all the symptoms of a severe grade of typhoid fever, which had commenced three days before his admission. He was put directly on the use of the solution of iodine, alternated with the turpentine and laudanum emulsion, to control a loose and tympanitic state of his bowels, with milk, and milk and flour gruel for nourishment. The case progressed favorably, and convalescence was established in about twenty days from the commencement of the disease, or seventeen days from the beginning of his treatment. This convalescence proceeded until he was able to be up, dressed, and about the house, with a good appetite and natural state of his evacuations. Nearly two weeks from the commencement of his convalescence, he began again to complain of the premonitory symptoms of fever, and on the third day had so distinct a chill that it was thought he might be under the influence of malaria, and he took three decigrams (grs. v) of quinine three times a day for two or three days. The fever, however, again assumed an unmistakable typhoid character, with considerable delirium and some looseness of the bowels. The original treatment with iodine and the emulsion was resumed, and has been continued to the present time. His temperature has declined below 38° C. (99° F.), in the morning, and 38.3° C. (101° F.), in the evening, with a clean, moist tongue, and other indications of approaching convalescence. I learn that this patient has formerly been affected with insanity, and his brain and nervous structures may have been somewhat enfeebled. But of the final results of his present sickness you will be informed in due time.

During the last four days three new cases of typhoid fever have been admitted into my wards, and all placed under the treatment I have been detailing. You can now examine them in their respective beds, and note their progress at subsequent clinic hours. To prevent being misunderstood, I will add, that I am not using iodine as a *specific* curative agent in typhoid fever, but simply as a general alterant and antiseptic, adapted to fulfill certain rational indications afforded by the pathology of the disease, and always to be aided by such collateral remedies as the abdominal or other local symptoms may indicate.

From my present experience, I am led to think it is a remedy of great value, especially when its use is commenced in the forming stage, or during the first week after the confinement of the patient from the development of the fever.

---

BORACIC ACID AS AN ANTISEPTIC IN CERTAIN SKIN AFFECTIONS.—Boracic acid being odorless and non-irritating, forms a convenient remedy in certain skin affections. By wearing stockings and cork soles saturated with boracic acid, the offensive odor from the feet is at once annihilated. Excessive perspiration of the same has also been permanently cured by boracic acid. An ointment of this is valuable in ulcerations, and should be expressly prepared in this manner: Make a solution of boracic acid in glycerine, and incorporate with a fatty basis of white wax and almond oil. It resembles cold cream. Vaseline is objectionable. Boracic acid is valuable in scrofulous ulcerations, abrasions, excoriations, etc.—(Extract from an article in *The Practitioner*, by G. Thin, M.D.)

